# Non-invasive detection of severe PH in lung disease using magnetic resonance imaging

**DOI:** 10.3389/fcvm.2023.1016994

**Published:** 2023-04-17

**Authors:** Dheyaa Alkhanfar, Krit Dwivedi, Faisal Alandejani, Yousef Shahin, Samer Alabed, Chris Johns, Pankaj Garg, A. A. Roger Thompson, Alexander M. K. Rothman, Abdul Hameed, Athanasios Charalampopoulos, Jim M. Wild, Robin Condliffe, David G. Kiely, Andrew J. Swift

**Affiliations:** ^1^ Department of Infection, Immunity and Cardiovascular Disease, University of Sheffield, Sheffield, United Kingdom; ^2^ INSIGNEO, Institute for in Silico Medicine, University of Sheffield, Sheffield, United Kingdom; ^3^ Department of Clinical Radiology, Sheffield Teaching Hospitals, Sheffield, United Kingdom; ^4^ Sheffield Pulmonary Vascular Disease Unit, Royal Hallamshire Hospital, Sheffield Teaching Hospitals NHS Foundation Trust, Sheffield, United Kingdom

**Keywords:** severe PH, chronic lung disease (CLD), COPD, ILD, cardiac MRI

## Abstract

**Introduction:**

Severe pulmonary hypertension (mean pulmonary artery pressure ≥35 mmHg) in chronic lung disease (PH-CLD) is associated with high mortality and morbidity. Data suggesting potential response to vasodilator therapy in patients with PH-CLD is emerging. The current diagnostic strategy utilises transthoracic Echocardiography (TTE), which can be technically challenging in some patients with advanced CLD. The aim of this study was to evaluate the diagnostic role of MRI models to diagnose severe PH in CLD.

**Methods:**

167 patients with CLD referred for suspected PH who underwent baseline cardiac MRI, pulmonary function tests and right heart catheterisation were identified. In a derivation cohort (*n* = 67) a bi-logistic regression model was developed to identify severe PH and compared to a previously published multiparameter model (Whitfield model), which is based on interventricular septal angle, ventricular mass index and diastolic pulmonary artery area. The model was evaluated in a test cohort.

**Results:**

The CLD-PH MRI model [= (−13.104) + (13.059 * VMI)—(0.237 * PA RAC) + (0.083 * Systolic Septal Angle)], had high accuracy in the test cohort (area under the ROC curve (0.91) (*p* < 0.0001), sensitivity 92.3%, specificity 70.2%, PPV 77.4%, and NPV 89.2%. The Whitfield model also had high accuracy in the test cohort (area under the ROC curve (0.92) (*p* < 0.0001), sensitivity 80.8%, specificity 87.2%, PPV 87.5%, and NPV 80.4%.

**Conclusion:**

The CLD-PH MRI model and Whitfield model have high accuracy to detect severe PH in CLD, and have strong prognostic value.

## Introduction

Group 3 Pulmonary Hypertension due to Chronic Lung Disease (PH-CLD) is associated with the worst prognosis amongst all forms of Pulmonary Hypertension (PH) (1). It is typically associated with mild-to-moderate PH and moderate to severe lung disease. Group 1 PH, pulmonary arterial hypertension (PAH), is typically associated with moderate-to-severe PH and minor or no lung disease. Patients however are increasingly identified with overlapping features and accurately classifying patients as PAH or PH-CLD is one of the most challenging areas in pulmonary vascular medicine (2). This is of particular clinical importance as the recommended management between PH-CLD and PAH is divergent. Only PAH patients are recommended to be treated with novel PAH specific drug therapies (3).

A particular group of interest is patients with PH-CLD who develop severe PH. Severe PH in the context of PH-CLD (Severe-PH-CLD) is defined as mean pulmonary artery pressure (mPAP) ≥ 35 mmHg or mPAP ≥25 mmHg with low cardiac index (CI <2.0 L/min/m^2^) (3–5). Whilst only a minority of patients with PH-CLD develop severe PH, given the prevalence of lung disease, this group is estimated to be far more common than PAH (5). Recent prospective data from two different registries have shown these patients have significantly poorer outcomes compared to those with non-severe disease (6–8).

Right Heart Catheterisation (RHC) is the gold standard for measurement of mPAP, but is an invasive test with a serious complication rate in inexperienced centres but with low morbidity and mortality in experienced centres (9). Echocardiography is the first-line screening test for elevated pulmonary artery pressure (PAP) because it is noninvasive, inexpensive, readily available, and portable, but has significant limitations, particularly in patients with severe lung disease. Estimation of systolic PAP was only possible in 44% of patients with severe lung disease due to poor acoustic windows, with 52% estimations found to be inaccurate (10). Echocardiographic estimation also requires the presence of tricuspid regurgitation (TR), which is not always present and severe TR causes erroneous results (11–13). However, other studies have demonstrated that some echocardiography formulas can accurately predict PH in CLD, including Chemla and Syyed formulas (14). The utility of alternate echocardiography measures were capable of detecting early right heart changes even before the development of severe PH (15). Also the combination of right ventricular systolic pressure (RVSP), right ventricular outflow tract (RVOT) diameter, and tricuspid annular plane systolic excursion (TAPSE) may be helpful in PH exclusion by the increased sensitivity and negative predictive value (16).

Magnetic resonance imaging is the gold standard to evaluate cardiac function and morphology (17). Multiparametric MRI diagnostic models have shown to correlate accurately with RHC and have high diagnostic accuracy in identifying patients with PH at both thresholds of >20 mmHg and ≥ 25 mmHg, but these models were not derived exclusively from patients with PH-CLD (13, 18).

The aim of this study was to develop and test CLD-PH MRI multivariate models that can identify Severe-PH-CLD. Non-invasive identification of these patients should improve clinical management and likely improve prognosis as there are new studies demonstrating the improvement of patients with PH due to interstitial lung disease on medical therapy such as treprostinil (19).

## Methods

### Patients

Patients undergoing systematic assessment for suspected PH were identified from the ASPIRE (Assessing the Severity of Pulmonary Hypertension In a Pulmonary Hypertension REferral Centre) registry between October 2012 and August 2019. Patients were required to have undergone a baseline assessment with magnetic resonance imaging, pulmonary function testing (PFT) and right heart catheterisation. All patients are assigned a diagnosis at a multidisciplinary team meeting.

**Figure 1:**
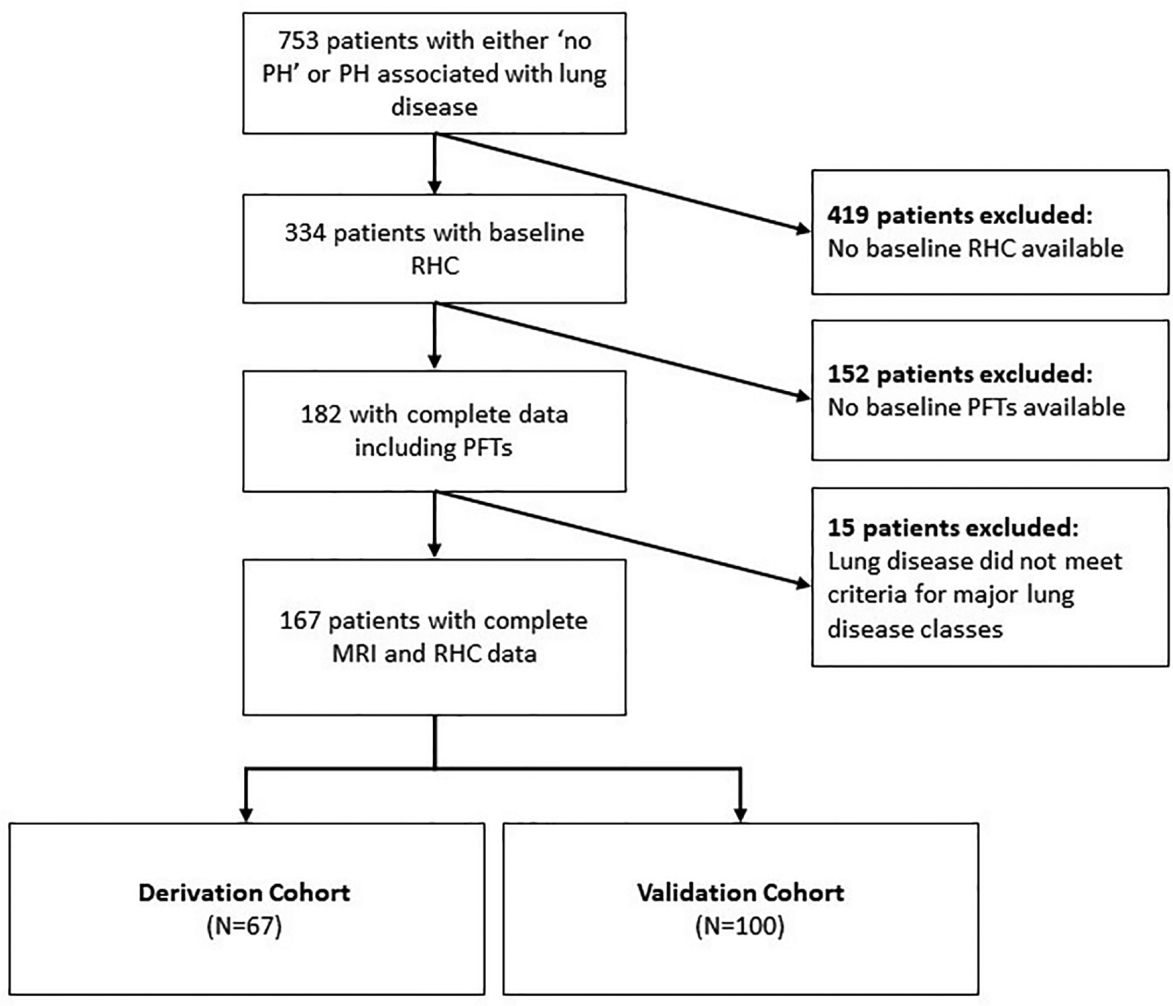
Patient selection flow chart. Inclusion criteria included those with complete RHC, PFT and MRI data and exclusion criteria included those with lung diseases not meeting criteria of major lung disease classes or those with coexisting disorders such as left heart disease. The classification of the derivation and test sets was according to the availability of their echocardiography data, where the derivation set of patients had no echocardiography measures. PH: pulmonary hypertension; RHC: right heart catheter; RFT: pulmonary function tests; MRI: magnetic resonance imaging.

Patients were classified into two groups, the derivation and test sets, according to the availability of their echocardiography data where the derivation set of patients had no echocardiography measures. Inclusion criteria required complete baseline MRI, PFT and RHC data (Figure 1). MRIs of the patients were performed within 24 h of the RHC. Patients with no RHC, no PFTs, and those with lung disease not meeting the criteria of major lung disease classes or have other coexisting conditions such as left heart disease were excluded. Approval for analysis of imaging data was granted by the local research ethics committee and consent was waived for this retrospective database study (ref c06/Q2308/8).

### MRI acquisition

MR imaging was performed with a 1.5-T HDx scanner. MRI was performed using the following techniques: an 8-channel cardiac coil on a GE HDx whole-body scanner (GE Healthcare, Milwaukee, WI), acquisition of short-axis cine images by utilising a cardiac gated multislice balanced SSFP sequence (twenty frames per each cardiac cycle, 8 mm slice thickness, field of view 48, matrix 256 × 256, BW 125 kHz/pixel, TR/TE 3.7/1.6 ms), a slice thickness of 8 mm with 2 mm inter-slice gaps were used to produce a stack of images in the short axis view to cover both ventricles from base to apex. The smallest cavity area was considered to be the end-systole. The first cine phase of the R-wave triggered acquisition or largest volume was considered to be the end-diastole. Through-plane, phase-contrast imaging was achieved orthogonal to the main pulmonary trunk. The parameters for the phase-contrast imaging included the following: slice thickness 10 mm, field of view 48 cm, bandwidth 62.5 kHz, matrix 256 X 128, repetition time TR 5.6 ms, echo time TE 2.7 ms, 20 reconstructed cardiac phases and velocity encoding of flow 150 cm/s. A surface coil was used on patients who were in the supine position with retrospective ECG gating (20).

### MR analysis

**Figure 2:**
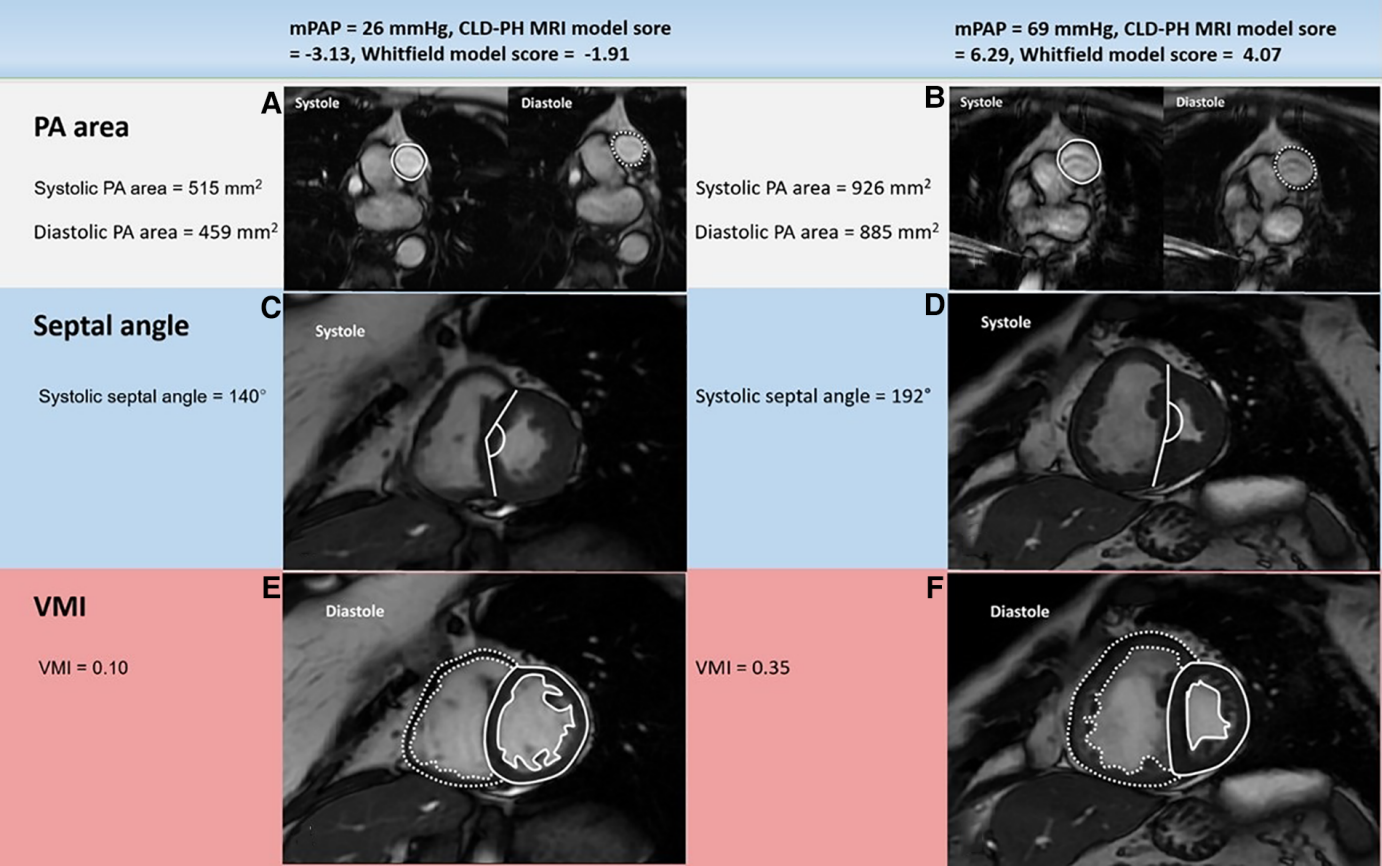
Example cardiac MRI images in patients with mPAP below (left) or above (right) 35 mmHg: PA area (**A, B**), systolic septal angle (**C, D**) and VMI (**E, F**). The patient with severe PH (right) had much larger systolic and diastolic PA areas, systolic septal curvature and VMI than the patient with mild-moderate PH (left). mPAP: mean pulmonary artery pressure; CLD: chronic lung disease; PH: pulmonary hypertension; PA: pulmonary artery; VMI: ventricular mass index; MRI: magnetic resonance imaging.

Image analysis was achieved using a GE Advantage Workstation 4.1 with the patient clinical information and the cardiac catheter data unavailable to the observer. Chambers trabeculations were included as part of the volume cavity measurement and were not separately traced (21). Right and left endocardial and epicardial surfaces were manually traced to obtain right ventricular end diastolic volume (RVEDV) and end systolic volume (RVESV), and left ventricular end diastolic volume (LVEDV) and end systolic volume (LVESV). Right ventricular ejection fraction (RVEF), left ventricular ejection fraction (LVEF), right ventricular stroke volume (RVSV) and left ventricular stroke volume (LVSV) were obtained using the end-diastolic and end-systolic volumes (20, 22, 23). These measurements were indexed for the body surface area and then corrected for age and sex and displayed as percentage predicted except RVEF and LVEF (24). SV was considered to be the most accurate from LV volumetry and was used to estimate RV-PA coupling and we have used the LV volumetry for SV rather than flow through PA/aorta as some studies proved the inaccuracy of the latter method and preferred the former method (25). Septal angle was measured as the angle of the septum from the RV insertion points to the centre of the mid septum (26). The interventricular septum was considered as part of the left ventricle for calculating the ventricular mass (Figure 2). RV end-diastolic mass (RV mass) and LV end-diastolic mass (LV mass) were derived. Ventricular mass index (VMI) was considered as the RV mass divided by the LV mass (27). Pulmonary artery (PA) areas were measured maximally and minimally and the following equation was used to calculate the relative area change: relative area change = (maximum area- minimum area)/minimum area (20). The well-established biplane area length method was used to measure the LA volume (28, 29).

### Transthoracic echocardiography

Clinically indicated transthoracic echocardiography (TTE) was performed according to local practice guidelines in the diagnostic assessment of the study patients. Systolic pulmonary arterial pressure was estimated from TR jet velocity and estimated right atrial pressure. An echocardiography threshold of systolic pulmonary artery pressure (sPAP) of 64 mmHg to predict severe PH was used (30, 31).

### Right heart catheterisation and ph severity

A balloon-tipped 7.5F thermodilution catheter (Franklin Lakes, Becton Dickinson, NJ) was inserted *via* the internal jugular vein to obtain RHC measurements including mPAP, pulmonary capillary wedge pressure (PAWP), and cardiac output (CO). The thermodilution technique was used to measure CO. Pulmonary vascular resistance (PVR) was defined as (mPAP-PAWP)/CO. Measurements of pressure were averaged during quiet breathing.

Our definitions are in the ESC/ERS guidelines on the management and treatment of PH rather than arbitrary thresholds (32). Severe Ph was classified as either i) mPAP ≥ 35 mmHg OR mPAP >25 and CI < 2l/min/m^2^ (5) or ii) Severe PH-CLD (mPAP > 20 mmHg, PVR > 400dynes/s/cm-5) (7). The latter was proposed by Zeder et al. as a non-biased approach to predict severe PH using PVR.

### Statistics

Statistical analysis was performed by using SPSS version 26.0 (SPSS, Chicago, Ill). A *p*-value <0.05 was considered significant. Histograms of MRI and clinical parameters were used to check normality and the data were normally distributed. An independent t-test was used to see the differences between the derivation and test cohorts, and the difference between patients with severe and mild-moderate PH. Binary logistic regression in the forward direction was performed to generate a multiparametric model for the prediction of severe PH in lung disease. The following variables were entered: age, sex, height, weight, forced expiratory volume in the first second (FEV1), forced vital capacity (FVC), systolic septal angle, LVEDV index, RVEDV index, RVESV index, RVEF, pulmonary artery relative are change (PA RAC) and VMI.

An MRI model was previously developed in a mixed cohort of patients with suspected PH using binary logistic regression to predict pulmonary hypertension: Whitfield model (arbitrary units) = −27.7 + 5.75loge(interventricular septal angle [degree of arc]) + 1.899loge(right ventricular mass/left ventricular mass) + 0.004 (diastolic pulmonary artery area [in square millimetres]) (18). The accuracy was compared against the model developed in the derivation cohort.

Pearson's correlation and linear regression between the derived regression equation model and Whitfield model for severe PH diagnosis and the mPAP measured by the RHC was calculated. The diagnostic performance for both models was assessed using ROC curves and AUC. Optimal diagnostic thresholds were identified in the derivation cohort using the Youden index and applied in the test cohort. Diagnostic accuracy was obtained from the 2 × 2 contingency table. Sensitivity, specificity, negative and positive predictive values were thus calculated from cross tabulation with their Pearson Chi-Square value and Fisher's exact test.

The follow-up period was considered the interval from MRI until all-cause death or census, the latter was performed on 01/02/2020. Survival analysis was accomplished using multivariate Cox proportional hazards regression in the forwards direction for the variables that were significantly associated with mortality in univariate regression. Kaplan-Meier plots were generated for the models, dichotomised by their threshold values, and Chi square values were calculated using the Log rank test.

## Results

167 patients met the inclusion criteria. The patients were divided into a derivation cohort (*n* = 67), and a test cohort (*n* = 100). Baseline characteristics for patients, grouped by mPAP ≥ 35 mmHg (severe PH, *n* = 90) and mPAP < 35 mmHg (no PH and mild-moderate PH, *n* = 77) are shown in Table 1. Patients with severe PH are older, have lower percent predicted DLCO, FEV1/FVC ratio, ISWT distance and poorer hemodynamics. There was no significant difference in gender, FVC and FEV1. As shown in Table 1, there were no significant differences in clinical demographic characteristics, RHC, PFTs nor MRI parameters between model derivation and test cohorts.

**Table 1:** Baseline demographics for all patients according to PH status and derivation or test cohort.

	No PH, Mild-moderate PH *n* = 77	Severe PH *n* = 90		Model development cohort *n* = 67	Test cohort *n* = 100	
Variables units	Mean (SD)	Mean (SD)	*P* value	Mean (SD)	Mean (SD)	*P* value
**Demographics**						
Age (years)	64 (12)	68 (11)	0.04	68 (11)	65 (12)	0.14
Sex female%	62%	52%	0.19	54%	59%	0.50
WHO functional class (I/II/III/IV) #	I (0) II (12) III (59) IV (3)	I (0) II (0) III (63) IV (27)	<0.001	I (0) II (8) III (45) IV (13)	I (0) II (4) III (77) IV (17)	0.32
Type of lung disease	Air trapping (*n*=5), COPD/emphysema (*n*=14), ILD/fibrosis (*n*=26), CPFE (*n*=5)	Air trapping (*n*=2), COPD/emphysema (*n*=44), ILD/fibrosis (*n*=30), CPFE (*n*=10)	** **	Air trapping (*n*=2), COPD/emphysema (*n*=19), ILD/fibrosis (*n*=23), CPFE (*n*=5)	Air trapping (*n*=1), COPD/emphysema (*n*=39), ILD/fibrosis (*n*=27), CPFE (*n*=4)	** **
**Right heart catheter data**						
mRAP (mmHg)	5 (3)	11 (6)	<0.001	9 (6)	8 (5)	0.39
mPAP (mmHg)	24 (6)	50 (8)	<0.001	39 (16)	37 (14)	0.43
PAWP (mmHg)	10 (4)	12 (7)	<0.001	11 (4)	11 (4)	0.81
Cardiac Output (L/min)	5.6 (2.2)	4.2 (1.6)	<0.001	4.7 (1.5)	5 (2.3)	0.33
Cardiac index (CI) (L/min/m^2^)	3.1 (1.1)	2.3 (0.8)	<0.001	2.5 (0.8)	2.7 (1.1)	0.14
PVR (Woods Unit)	226 (107)	817 (387)	<0.001	576 (442)	515 (395)	0.35
SaO_2_ (%)	96 (3)	92 (6)	<0.001	94 (5)	94 (5)	0.94
SVO_2_ (%)	70 (7)	62 (8)	<0.001	66 (9)	66 (8)	0.82
**ISWT—distance (m)**	219 (169)	112 (89)	<0.001	153 (132)	168 (149)	0.50
**Pulmonary Function Tests (PFTs)**						
Percent predicted FEV^1^ (%)	70.65 (21.99)	65.10 (19.16)	0.08	66.46 (21.71)	68.46 (19.96)	0.54
Percent predicted FVC (%)	78.49 (23.43)	78.82 (20.05)	0.92	77.22 (24.28)	79.63 (19.69)	0.48
FEV^1^/FVC ratio (%)	0.92 (0.20)	0.84 (0.20)	0.01	0.88 (0.20)	0.87 (0.21)	0.80
Percent predicted DLCO (%)	47.62 (20.16)	27.22 (13.05)	<0.001	34.49 (20.23)	37.61 (18.91)	0.32
**MRI parameters**						
Time between RHC and MRI (d)	0 (2)	0 (1)	0.43	0 (2)	0 (0)	0.44
LVEDV index (ml/m^2^)	59.30 (14.86)	47.66 (15.72)	<0.001	51.53 (16.64)	54.03 (16.16)	0.33
LVSV index (ml/m^2^)	39.64 (10.52)	30.58 (10.88)	<0.001	33.42 (35.65)	11.28 (11.79)	0.22
RVESV index (ml/m^2^)	35.80 (16.86)	59.72 (26.98)	<0.001	47.53 (23.20)	49.47 (27.42)	0.63
RVEF (%)	47.57 (10.79)	35.70 (11.81)	<0.001	42.52 (12.13)	40.27 (13.18)	0.26
RV systolic mass	30.68 (17.47)	56.40 (31.38)	<0.001	41.94 (22.34)	47.14 (33.14)	0.32
Diastolic PA area (mm^2^)	676.11 (191.11)	893.71 (246.59)	<0.001	821.51 (275.57)	773.33 (225.35)	0.21
Systolic PA area (mm^2^)	765.07 (214.63)	977.93 (273.48)	<0.001	909.02 (303.68)	859.01 (242.57)	0.24
PA RAC (%)	13.90 (7.27)	9.26 (4.26)	<0.001	10.89 (5.98)	11.84 (6.52)	0.37
VMI	0.17 (0.09)	0.31 (0.16)	<0.001	0.25 (0.14)	0.25 (0.15)	0.94
Systolic septal angle (°)	144.70 (14.68)	175.47 (18.18)	<0.001	162.61 (24.01)	160.40 (21.75)	0.53

WHO, world health organisation, mRAP, mean right atrial pressure, mPAP, mean pulmonary arterial pressure, PAWP, pulmonary artery wedge pressure, PVR, pulmonary vascular resistance, SaO_2_, Oxygen saturation, SVO_2_, Mixed venous oxygen saturation, FEV^1^, Forced Expiratory Volume in 1 s, FVC, Forced Vital Capacity, DLCO, transfer capacity of the lung for the uptake of carbon monoxide (CO), RVESV, right ventricular end-systolic volume; RVEF, right ventricular ejection fraction; LVEDV, left ventricular end-diastolic volume; LVSV, left ventricular stroke volume; VMI, ventricular mass index; PA RAC, pulmonary artery relative area change; PA, pulmonary artery; RV, right ventricle.

WHO functional class data is missing for 2 patients.

### Correlation between MRI features and mPAP

The strongest correlations with mPAP were for systolic septal angle (*r*=0.74), DLCO (*r*=−0.56), RVEF (*r*=−0.53), RVESV index and systolic PA area (both *r*=0.46) and VMI (*r*=0.45). PA relative area change, LVEDV index, RV systolic mass, diastolic PA area and systolic PA area all also showed significant correlations with mPAP.

### Regression analysis

Regression analysis produced the following equation predictive of elevated mPAP ≥ 35 mmHg: CLD-PH MRI model=(-13.104)+(13.059 * VMI)—(0.237 * PA RAC)+(0.083 * systolic septal angle).

### Diagnostic threshold identification

Severe PH (*mPAP* ≥ *35 mmHg OR mPAP >25 and CI < 2l/min/m^2^*): The CLD-PH MRI model's highest Youden index point was 0.77, with a sensitivity of 95% and specificity of 83%, giving a threshold of −0.75. For the Whitfield model, the highest Youden index point was 0.74 with a sensitivity of 85% and specificity of 88%. A value greater than 1.66 was identified as optimally diagnostic.

*Severe PH-CLD (mPAP > 20 mmHg, PVR > 400 dynes/s/cm^5^):* The CLD-PH MRI model's highest Youden index was 0.74 with a sensitivity of 92% and specificity of 82% and a value greater than −0.75 was optimally diagnostic. For the Whitfield model, the highest Youden index point was 0.71, with a sensitivity of 92% and specificity of 78%, giving a threshold of 1.05.

## Test cohort diagnostics

### Agreement

The CLD-PH MRI model generated from the derivation cohort correlated strongly with RHC-measured mPAP (*r*=0.71). There is a moderate intraclass correlation for the estimation of mPAP of 0.53. The Whitfield's model also correlated strongly with the RHC-measured mPAP (*r*=0.73) with a moderate intraclass correlation of 0.51 for mPAP estimation.

### Diagnostic accuracy

*-Severe PH (mPAP* ≥ *35 mmHg OR mPAP >25 and CI < 2l/min/m^2^):*

**Figure 3:**
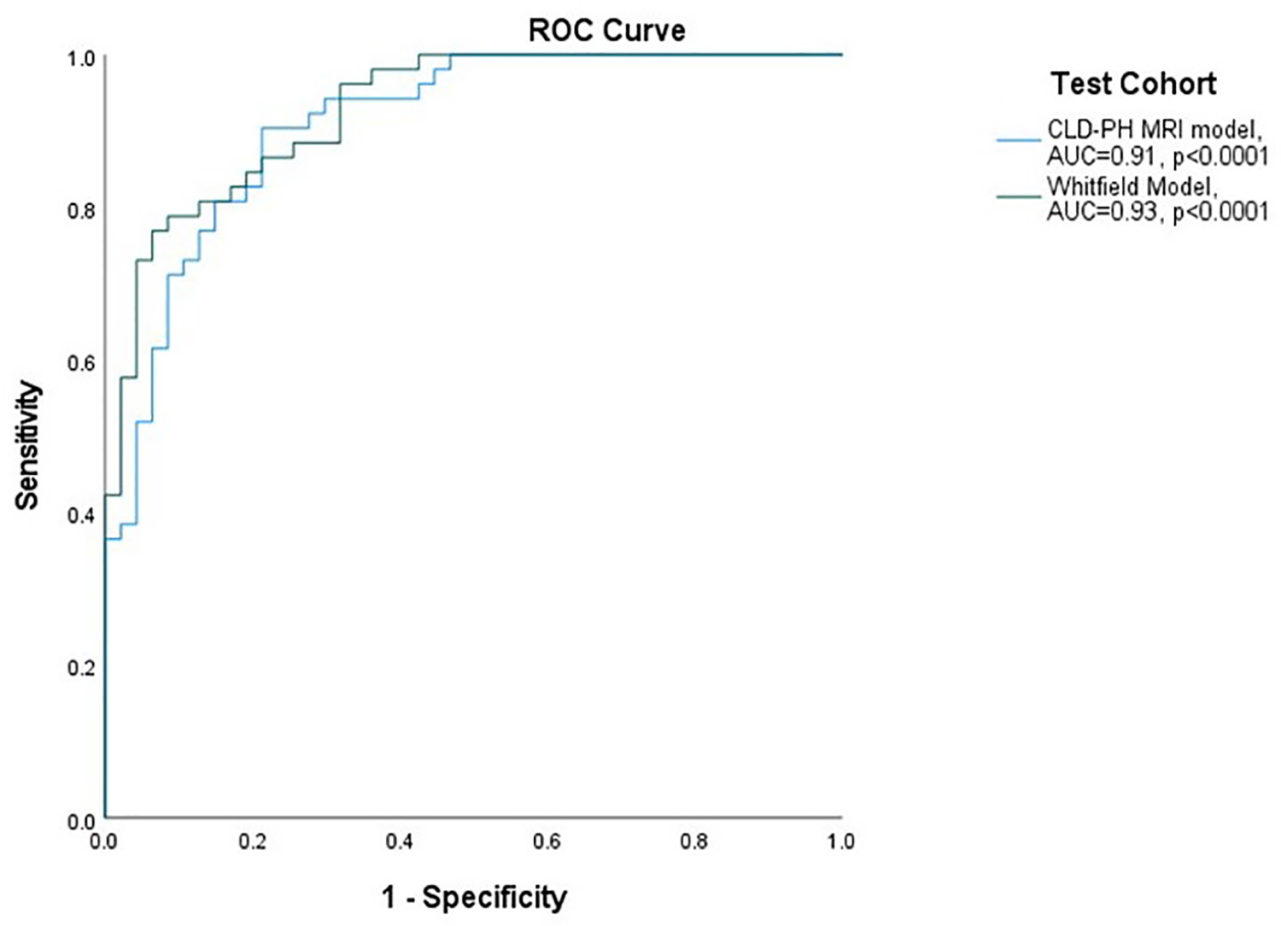
ROC curves for performance of models in severe PH (mPAP ≥ 35 mmHg OR mPAP >25 and CI < 2l/min/m^2^). ROC: receiver operating characteristic curve; AUC: area under the ROC curve; mPAP: mean pulmonary artery pressure; CI: cardiac index; CLD: chronic lung disease; PH: pulmonary hypertension; MRI: magnetic resonance imaging.

The **CLD-PH MRI model** showed high diagnostic accuracy; area under the ROC curve 0.91 and *p* < 0.0001 (Figure 3), with sensitivity 92.3%, specificity 70.2%, PPV 77.4% and NPV 89.2% (Table 2). Pearson Chi-Square value was 41.22 and Fisher's exact test value was <0.0001.

**Table 2:** Models diagnostic performance in the test cohort to predict severe PH (mPAP ≥ 35 mmHg OR mPAP >25 and CI < 2l/min/m2).

Parameter	MRI model (*n*=99)	Whitfield model (*n*=99)	Echo sPAP (*n*=68)
Correlation with RHC-measured mPAP	0.71	0.73	0.64
ICC with mPAP	0.53	0.51	0.13
Sensitivity (%)	92.3%	80.8%	63.2%
Specificity (%)	70.2%	87.2%	86.7%
Positive predictive value (%)	77.4%	87.5%	85.7%
Negative predictive value (%)	89.2%	80.4%	65.0%
AUC	0.91	0.93	0.88

AUC, area under the receiver operating characteristic curve, ICC, intraclass correlation coefficient, mPAP, mean pulmonary arterial pressure, RHC, right-sided heart catheterisation; PH: pulmonary hypertension; CI: cardiac index.

**Whitfield model** in test cohort: area under the ROC curve was 0.93 and *p* < 0.0001 (Figure 3), with sensitivity 80.8%, specificity 87.2%, PPV 87.5%, NPV 80.4% (Table 2). Pearson Chi-Square value for the test was 45.71 and Fisher's exact test value was <0.0001.

Table 3 shows the models diagnostic performance in the test cohort to predict severe PH stratified by lung disease class.

**Table 3:** Models diagnostic performance in the test cohort to predict severe PH (mPAP ≥ 35 mmHg OR mPAP >25 and CI < 2l/min/m^2^) stratified by lung disease class.

A. COPD/Emphysema in test cohort			
Parameter	MRI model (*n*=40)	Whitfield model (*n*=40)	Echo sPAP (*n*=29)
Correlation with RHC-measured mPAP	0.71	0.69	0.56
ICC with mPAP	0.60	0.38	0.64
Sensitivity (%)	96.6%	82.8%	61.9%
Specificity (%)	63.6%	100.0%	87.5%
Positive predictive value (%)	87.5%	100.0%	92.9%
Negative predictive value (%)	87.5%	68.8%	46.7%
AUC	0.96	0.93	0.83
(B) ILD in test cohort			

Parameter	MRI model (*n*=43)	Whitfield model (*n*=43)	Echo sPAP (*n*=32)
Correlation with RHC-measured mPAP	0.706	0.708	0.711
ICC with mPAP	0.55	0.34	0.78
Sensitivity (%)	93.3%	80%	71.4%
Specificity (%)	78.6%	85.7%	88.9%
Positive predictive value (%)	70%	75%	83.3%
Negative predictive value (%)	95.7%	88.9%	80%
AUC	0.87	0.89	0.95

AUC, area under the receiver operating characteristic curve, ICC, intraclass correlation coefficient, mPAP, mean pulmonary arterial pressure, RHC, right-sided heart catheterisation; COPD: chronic obstructive pulmonary disease; ILD: interstitial lung disease.

**Systolic pulmonary artery pressure (sPAP)** could be estimated in 68 patients who underwent Echocardiography in the test cohort. 32 patients (32%) had no recorded sPAP.

Echo threshold. The diagnostic value of sPAP 64 mmHg was: area under the ROC curve was 0.88 and *p* < 0.0001, with sensitivity 63.2%, specificity 86.7%, PPV 85.7%,and NPV 65.0% (Table 2). Pearson Chi-Square value for the test was 17.18 and Fisher's exact test value was <0.0001.

*-Severe PH-CLD (mPAP > 20 mmHg, PVR > 400dynes/s/cm-5):*

The **CLD-PH MRI model** revealed a high diagnostic accuracy; area under the ROC curve 0.90, *p* < 0.0001, with sensitivity of 96.1%, specificity 72.9%, PPV 79% and NPV 94.6% (Supplementary Table 1). Pearson Chi-Square value was 50.29 and Fisher's exact test value was <0.0001.

**Whitfield model** showed also a high diagnostic accuracy; AUC ROC 0.92 and *p* < 0.0001, with sensitivity 88.2%, PPV 80.4% specificity 77.1%, NPV 86% (Supplementary Table 1). Pearson Chi-Square value for the test was 42.94 and Fisher's exact test value was <0.0001.

The diagnostic value of **sPAP** 64mmHg was: area under the ROC curve 0.84 and *p* < 0.0001, with sensitivity 63.9%, specificity %84.4%, PPV 82.1%,and NPV 67.5% (Supplementary Table 1). Pearson Chi-Square value for the test was 16.29 and Fisher's exact test value was <0.0001.

Supplementary table 2 demonstrates models diagnostic performance in the test cohort to predict severe PH stratified by lung disease class.

**Prognostic value in full cohort:**

*mPAP* ≥ *35 mmHg OR mPAP >25 and CI < 2l/min/m^2^*

**Figure 4:**
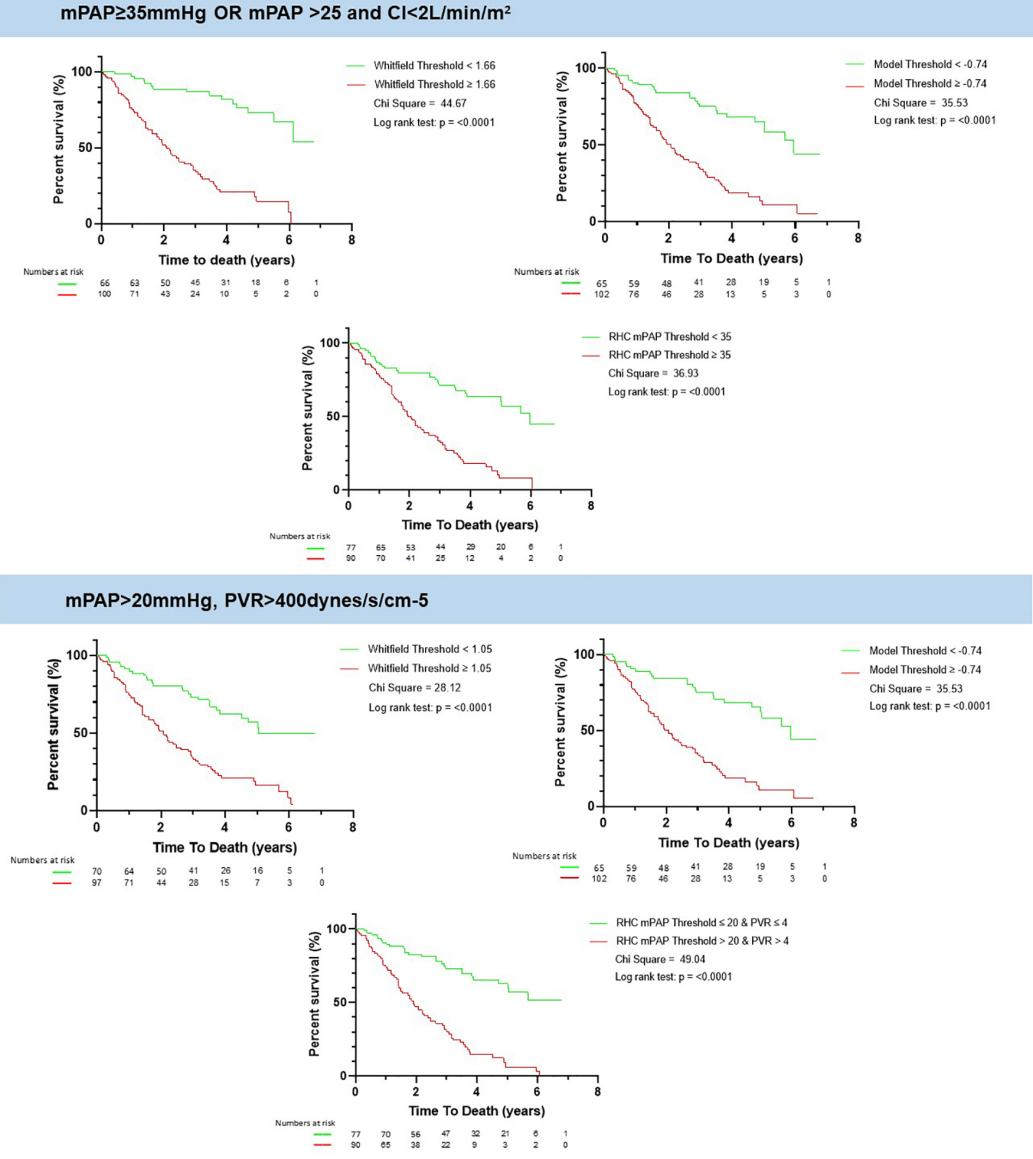
Kaplan meier of whitfield and lung disease CLD-PH MRI model and the RHC measured mPAP. There is an increased mortality above the selected thresholds of Whitfield model, CLD-PH MRI model and RHC-measured mPAP for both definitions of severe PH according to the ESC/ERS guidelines. mPAP: mean pulmonary artery pressure; PVR: pulmonary vascular resistance; CI: cardiac index; CLD: chronic lung disease; PH: pulmonary hypertension; RHC: right heart catheter; MRI: magnetic resonance imaging.

A value of the CLD-PH MRI model ≥ −0.74 was associated with worse survival than patients with model value less than −0.74, log rank chi square 35.53, *p* < 0.0001, Figure 4. The same patients with a value of the Whitfield model ≥1.6 had worse survival than those less than 1.6, log rank chi square 44.47, *p* < 0.0001. A value of RHC-measured mPAP ≥ 35 mmHg was associated with worse survival than those less than 35 mmHg, log rank chi square 36.93, *p* < 0.0001.

*-Severe PH-CLD (mPAP > 20 mmHg, PVR > 400dynes/s/cm-5):*

A value of the CLD-PH MRI model ≥ −0.74 was associated with worse survival than patients with model value less than −0.74, log rank chi square 35.53, *p* < 0.0001, Figure 4. The same patients with a value of the Whitfield model ≥1.05 had worse survival than those less than 1.05, log rank chi square 28.12, *p* < 0.0001. A value of RHC-measured mPAP > 20 mmg was associated with worse survival than those equal or less than 20 mmHg, log rank chi square 49.04, *p* < 0.0001.

On Cox regression, adjusting for age, sex and body surface area (BSA), the CLD-PH MRI model remained a statistically significant predictor of mortality (hazard ratio 1.22; 95% CI: 1.13, 1.32, *p* < 0.0001). The Whitfield model also remained significant (hazard ratio=1.28; 95% CI: 1.17, 1.41, *p* < 0.0001).

## Discussion

We have derived a multivariate binary logistic regression model and have shown it has a high diagnostic accuracy identifying a threshold to diagnose severe PH-CLD in a test cohort. The model further provides prognostic information similar to RHC derived mPAP. Given the challenge of echocardiography in patients with severe lung disease, MRI may play an important role in diagnosis, and this study shows that in patients not positively diagnosed by high estimated sPAP at Echocardiography, MRI can diagnose severe PH with high accuracy.

There is active research interest in sub-phenotyping PH-CLD and identifying groups of patients in whom PAH therapy is of benefit. Severe PH-CLD is an important potential subgroup, as these patients are characterised with moderate airway obstruction, but marked dyspnea, hypoxemia, low DLCO, high mPAP, and poor prognosis (6). Recent studies have affirmed the utility of hemodynamic measurements in identifying patients with poor prognosis. Zeder (7) recently demonstrated PVR to be the strongest predictor of poor survival in Severe-PH-CLD and COPD, with a prognostic cut-off of 5 WU, after adjusting for age, sex and FEV1. Olsson similarly found a PVR >5 to be associated with significantly worse survival, but in the context of ILD, a large cohort from the COMPERA registry (8).

Historically multiple randomised controlled trials have investigated different therapeutic agents in PH-CLD as a whole against a variety of endpoints, with varying results and mixed evidence for therapeutic response (2, 33–38). However, recently the INCREASE study demonstrated an improvement in 6MWD, NT-proBNP and clinical worsening in PH-CLD patients with ILD treated with inhaled treprostinil (19). The median mPAP for these patients was high (37.2 mmHg). Subgroup analysis revealed these beneficial effects were only seen in patients with a PVR ≥4 WU (2, 19). A post-hoc analysis of INCREASE showing inhaled treprostinil was associated with improved FVC vs. placebo (33).

These studies highlight a need to carefully sub-group patients, to aid in identification of phenotypes which may demonstrate therapy response. This study enables identification of severe PH-CLD using cardiac MRI, which is the established gold standard for anatomical and functional assessment.

Cumulative findings in multimodality imaging in PH are of paramount importance in screening, diagnosis, management and prognosis of patients with PH (39). Our proposed multivariate model is composed of: i) measurements of displacement of interventricular septum which is a marker of volume and pressure differential between the left and right ventricle, ii) ventricular mass index which is a marker for right ventricular remodelling, and iii) pulmonary artery relative area change which is a marker of stiffness of proximal pulmonary vasculature (40). The MRI measurements are easily measured from the standard MRI sequences, take little time to process and are reproducible (20).

It has been shown that septal angle, which correlated strongly with RHC-measured mPAP, improves the diagnostic accuracy of PH (26), additionally, the inclusion of VMI with a measure of septal angle has been proven to increase the accuracy of PH diagnosis (41), which complies with our study. The value of computational models of pulmonary arterial flow has also been assessed for the diagnosis of PH (42). PA RAC has been strongly linked to mortality and long term outcome in some studies (43).

Prediction models have been widely developed in recent years. A stepwise echocardiographic score to detect severe PH (mPAP⩾35 mmHg) was developed and validated by utilising tricuspid regurgitant velocity and right ventricular systolic pressure (right atrial pressure) and additional echocardiographic signs (30). Another novel echocardiography scoring system was developed and showed high capacity for predicting severe PH-CLD which implies the value of non-invasive examinations (44). The model developed in this study has a higher sensitivity but lower specificity compared to the prior Whitfield model. This may be more appropriate in the context of screening; the added value of this study is that the CLD-PH MRI model is specifically trained on severe PH and further studies that apply such MRI models in clinical practice compared to the gold standard (RHC) are warranted. Echocardiography in our cohort shows good accuracy for the diagnosis of PH, however we noted that there are groups of patients with severe lung disease for whom sPAP assessment is not achievable. Hence for these patients MRI may be a viable alternative.

The new MRI model does have higher sensitivity so may lend itself better to screening of patients with lung disease rather than more definitive predictions that are required at the tertiary centre. The Whitfield model has not previously been tested in this patient's population and has not been used to predict severe PH previously, hence the data on the Whitfield model and the new MRI model add new information to the literature. Echo is the least sensitive, though a limitation is that a full execution of the ESC/ERS guidelines of Echo was not possible in this study.

This work adds to the literature in that a new model has been developed that may have additional clinical value given the high sensitivity to detect the disease. Testing the Whitfield model in this population has also added new knowledge to the field, and has been shown to be able to diagnose severe PH in lung disease in addition to diagnosis of “suspected pulmonary hypertension” as previously described.

### Limitations

The study is limited by its retrospective and single tertiary specialist referral centre design. Further validity of the model can be assessed by applying the model in other PH centres and centres with low prevalence of disease. A larger prospective study would compare application of the MRI model against the current gold standard of RHC would add further validity to the findings. Quantitative Echocardiography data from the right ventricle was not available and thus we were unable to fully employ the “other signs” as per the ERS/ERS guidelines. One of the limitations of MRI as a clinical tool is that the role of MRI may be as a second line test in patients with suspected severe PH in lung disease in addition to other disadvantages of MRI being not readily available, expensive and also MRI is poorly tolerated in patients with severe lung disease who are oxygen dependent who may struggle with breath-holding.

## Conclusion

A model consisting of easy-to-obtain cardiac MRI metrics can facilitate the detection of severe PH in lung disease with high accuracy. This approach further allows identification of those patients at high risk of mortality.

## Data Availability

The raw data supporting the conclusions of this article will be made available by the authors, without undue reservation.
