# Supplementary material for: Non-invasive detection of severe PH in lung disease using magnetic resonance imaging
Source: Front Cardiovasc Med. 2023 Apr 17;10:1016994. doi: 10.3389/fcvm.2023.1016994 (PMC10149807; doi:10.3389/fcvm.2023.1016994)
Supplement: Supplementary file 1 [file Table1.docx]

**Supplementary table 1:** Models diagnostic performance in the test cohort to predict severe PH (mPAP>20mmHg, PVR>400dynes/s/cm-5).

| **Parameter** | **MRI model (n=99)** | **Whitfield model (n=99)** | **Echo sPAP (n=68)** |
| --- | --- | --- | --- |
| Correlation with RHC-measured mPAP | 0.71 | 0.73 | 0.64 |
| ICC with mPAP | 0.53 | 0.51 | 0.13 |
| Sensitivity (%) | 96.1% | 88.2% | 63.9% |
| Specificity (%) | 72.9% | 77.1% | 84.4% |
| Positive predictive value (%) | 79% | 80.4% | 82.1% |
| Negative predictive value (%) | 94.6% | 86% | 67.5% |
| AUC | 0.90 | 0.92 | 0.84 |

AUC = area under the receiver operating characteristic curve, ICC = intraclass correlation coefficient, mPAP = mean pulmonary arterial pressure, RHC = right-sided heart catheterisation; PVR: pulmonary vascular resistance.

**Supplementary table 2:** Models diagnostic performance in the test cohort to predict severe PH (mPAP>20mmHg, PVR>400dynes/s/cm-5) stratified by lung disease class.

1. COPD/Emphysema in test cohort

| **Parameter** | **MRI model (n=40)** | **Whitfield model (n=40)** | **Echo sPAP (n=29)** |
| --- | --- | --- | --- |
| Correlation with RHC-measured mPAP | 0.71 | 0.69 | 0.56 |
| ICC with mPAP | 0.60 | 0.38 | 0.64 |
| Sensitivity (%) | 100% | 85.2% | 68.4% |
| Specificity (%) | 61.5% | 76.9% | 90% |
| Positive predictive value (%) | 84.4% | 88.5% | 92.9% |
| Negative predictive value (%) | 100% | 71.4% | 60% |
| AUC | 0.86 | 0.84 | 0.80 |

1. ILD in test cohort

| **Parameter** | **MRI model (n=43)** | **Whitfield model (n=43)** | **Echo sPAP (n=32)** |
| --- | --- | --- | --- |
| Correlation with RHC-measured mPAP | 0.706 | 0.708 | 0.711 |
| ICC with mPAP | 0.55 | 0.34 | 0.78 |
| Sensitivity (%) | 94.1% | 94.1% | 69% |
| Specificity (%) | 84.6% | 80.8% | 82.4% |
| Positive predictive value (%) | 80% | 76.2% | 75% |
| Negative predictive value (%) | 95.7% | 95.5% | 70% |
| AUC | 0.89 | 0.93 | 0.86 |

AUC = area under the receiver operating characteristic curve, ICC = intraclass correlation coefficient, mPAP = mean pulmonary arterial pressure, RHC = right-sided heart catheterisation; COPD: chronic obstructive pulmonary disease; ILD: interstitial lung disease.
